# Antimicrobial metabolite profiling of *Nigrospora sphaerica* from *Adiantum philippense* L.

**DOI:** 10.1186/s43141-020-00080-4

**Published:** 2020-10-22

**Authors:** Kolathuru Puttamadaiah Ramesha, Nagabhushana Chandra Mohana, Bettadapura Rameshgowda Nuthan, Devaraju Rakshith, Sreedharamurthy Satish

**Affiliations:** Microbial Drug Technological Laboratory, Department of Studies in Microbiology, University of Mysore, Manasagangotri, Mysore, Karnataka 570006 India

**Keywords:** *Adiantum philippense*, *Nigrospora sphaerica*, Phomalactone, Antimicrobial activity, TLC-bioautography

## Abstract

**Background:**

Endophyte bestows beneficial aspects to its inhabiting host, along with a contribution to diverse structural attributes with biological potential. In this regard, antimicrobial profiling of fungal endophytes from medicinal plant *Adiantum philippense* revealed bioactive *Nigrospora sphaerica* from the leaf segment. Chemical and biological profiling through TLC–bioautography and hyphenated spectroscopic techniques confirmed the presence of phomalactone as an antimicrobial metabolite.

**Results:**

The chemical investigation of the broth extract by bioassay-guided fractionation confirmed phomalactone as a bioactive antimicrobial secondary metabolite. The antimicrobial activity of phomalactone was found to be highest against *Escherichia coli* by disc diffusion assay. The MIC was found to be significant against both *Escherichia coli* and *Xanthomonas campestris* in the case of bacteria and dermatophyte *Candida albicans* at 150 μg/ml, respectively.

**Conclusions:**

Overall, the results highlighted the antimicrobial potential of phomalactone from the endophyte *Nigrospora sphaerica* exhibiting a broad spectrum of antimicrobial activity against human and phytopathogenic bacteria and fungi. This work is the first report regarding the antibacterial activity of phomalactone.

## Background

Endophytes such as bacteria, fungi, and actinomycetes inhabit healthy plant tissues and have a crucial role in the defense mechanism of the host against pathogens, herbivores, and abiotic stresses [27, 35]. Until the previous decade, research on endophytic microbes was confined only to diversity and distribution. However, a new avenue was envisioned because of taxol from *Taxomyces andreanae* inhabiting *Taxus brevifolia* [18, 26].

Natural products from endophytes have gained wide attention from the research community due to their wide structural diversities with potential biological applications. However, the quest for chemical diversity attributed to secondary metabolites from endophytes has been led to an increased number of redundancies; making it laborious for natural product researchers for confining new analogs with potent biological activities [16, 24].

The genus *Nigrospora* has been reported previously to produce bioactives viz., nigrosporolides, phomalactone [8, 10], nigrosporins [17], lactones, epoxydons [29], diterpenes, diketopiperazines, lactones [25], nigrosporolides, and pyrones. The plant source *Adiantum philippense* L. is commonly known as “Hanspadi” or '”Strolling Maidenhair plant.” *A. philippense* has been traditionally used numerous parts of India as an ethnomedicinal as well as the cosmetic plant [21]. The ethnomedicinal aspects include the treatment of illnesses, consuming sensation, erysipelas, epileptic fits, looseness of the bowels, bronchitis, coughing, and elephantiasis [2]. Earlier reports have stated the potential capacity of the plant exhibiting antibacterial and antifungal activities.

The present investigation emphasizes the identification of endophytic fungus *N. sphaerica* inhabiting *A. philippense*, and bioactivity-guided dereplication process led to the isolation of a potent antimicrobial agent phomalactone. To the best of our knowledge, this is the first report elaborating the antibacterial activity of phomalactone.

## Methods

### Materials

Potato dextrose agar (PDA) and broth (PDB), Mueller-Hinton agar (MHA) and broth (MHB), Sabouraud dextrose agar (SDA), gentamicin, nystatin, and sodium dodecyl sulfate (SDS) were purchased from HiMedia (Mumbai, India). Other materials were obtained from different manufacturers such as sodium hypochlorite solution (available chlorine 4%) from Fisher Scientific (Mumbai, India); Precoated aluminum-backed TLC silica gel plates (60 F_254_) from Merck (Darmstadt, Germany); HPLC grade solvents like ethyl acetate; hexane and methanol from SDFCL (Mumbai, India); and Silica gel 60–120 mesh from Qualigens (Mumbai, India). The ITS 1 and ITS 4 primers, 2,3,5-triphenyl tetrazolium chloride (TTC), and Thiazolyl Blue Tetrazolium Bromide (MTT) were procured from Sigma-Aldrich (Missouri, USA).

#### Test organisms

All the test microbial pathogens such as *Staphylococcus aureus* (MTCC 7443), *Bacillus subtilis* (MTCC 121), *Bacillus cereus* (MTCC 430), *Staphylococcus epidermidis* (MTCC 435), *Escherichia coli* (MTCC 7410), *Klebsiella pneumonia* (MTCC 7407), *Salmonella typhi* (MTCC 733), *Shigella flexneri* (MTCC 1457), *Vibrio parahaemolyticus* ((MTCC 451), *Xanthomonas campestris* (MTCC 7908), and *Candida albicans* (MTCC 183) were procured from Microbial Type Culture Collection and Gene Bank (MTCC; Chandigarh, India).

#### Plant collection and isolation of fungal endophytes

Healthy asymptomatic *A. philippense* was collected from the Western Ghats region near Virajpete (12.2° N, 75.8° E). The plant specimen was identified by Dr. S. Mahadevakumar (field taxonomist) and submitted to herbarium (Voucher specimen number: UOM-BOT20-AP02). The plant material was sealed in sterile polythene bags and transported to the lab and processed within 8 h of collection. The plant material was initially washed under tap water for removing unwanted debris and soil; followed by sterile double distilled water (Dw) washes for twice. The plant leaf material was separated and cut into segments of 0.5 cm^2^ size. The plant segments were subjected to surface sterilization with 75% (*v/v*) ethanol (1 min), followed by 4% (*v/v*) NaOCl (4 min) and 75% (*v/v*) ethanol (1 min). Finally, tissue bits were rinsed with sterile double distilled water to remove residual surface sterilizing agents and blot dried under aseptic conditions. The surface-sterilized and blot dried leaf segments were placed on potato dextrose agar amended with antibiotic chloramphenicol (100 mg/L) to eliminate bacterial growth followed by incubation in a light chamber for two weeks at a 12-h light/dark cycles at 25 ± 2 °C. The fungal colonies were picked and transferred onto fresh potato dextrose agar plates devoid of any antibiotics [15, 23].

#### Genomic DNA extraction

Bioactive mycoendophyte *N. sphaerica* isolated from *A. philippense* was cultured in potato dextrose broth for a week at 25 ± 2 °C for isolation of genomic DNA. The mycelial mat was harvested, grounded, and transferred to a microcentrifuge tube containing 1 ml of 2× CTAB extraction buffer and incubated at 65 °C water bath for 30 min, later centrifuged at 10,000 g (10 min; RT). The aqueous phase was mixed with equal volume phenol: chloroform: isoamyl alcohol (25:24:1) for total DNA extraction followed by the addition of propanol to precipitate DNA [9].

#### Polymerase chain reaction amplification

The PCR amplification was performed according to the protocol of White et al. [31] using ITS1 and ITS4 set of universal primers. The PCR reaction mixture (50 μL) was prepared with 5 μL PCR buffer (10X; 15 mM MgCl_2_), 5 μL of dNTPs (2 mM), 2 μL of forward and reverse primers (5 pmole/μL), 2 μL Taq polymerase (1 U/μL), and the final volume was made up for 50 μL using Nanopure water (30 μL). The PCR reaction was carried out for 30 cycles with the following conditions denaturation 94 °C (40 s), annealing at 54 °C (60 s), extension at 72 °C (60 s), and final extension for a 10-min interval at 72 °C. The sequence was analyzed using ABI 3730 sequencer.

#### Antimicrobial screening by agar plug method

The pure cultures of fungal endophytes isolated from *A. philippense* were preliminarily screened for antimicrobial activity using the agar plug diffusion method. Pure cultures of fungal endophytes were cultured in potato dextrose agar for 21 days at 25 ± 2 °C. The agar plugs of pure cultures were placed onto Mueller-Hinton agar preseeded with test organisms, followed by refrigeration at 4 °C for 1 h to facilitate diffusion of metabolites, and further plates were incubated at 37 ± 2 °C (24 h) for bacteria and 25 ± 2 °C (72 h) for fungi. After incubation, zones of inhibition around the agar plug were observed [37].

#### Fermentation and extraction of secondary metabolites

Bioactive *N. sphaerica* was cultured in 2 L Erlenmeyer flasks (10) containing 450 ml potato dextrose broth at 25 ± 2 °C for 4 weeks under static conditions. The culture broth was filtered, and the liquid supernatant was extracted thrice with an equal volume of ethyl acetate and concentrated using a rotary flash evaporator (Rotavapor R® R-3, Buchi) (50 °C) and it was stored at 4 °C. The ethyl acetate extract concentrate was fractionated by column chromatography over a silica gel column (50 g, 60–120 mesh) using stepwise gradient elution from hexane: ethyl acetate (100:0 to 0:100) mixtures with increasing polarity, to afford different fractions (eluates). The fractions obtained were developed in TLC for pooling the similar fractions using optimized solvent system ethyl acetate: hexane (1:1; *v/v*) [22].

#### Detection of antimicrobial metabolite by TLC-bioautographic assay

Analytical TLC was employed for detecting antimicrobial metabolites. Ten microliters of flash evaporated ethyl acetate fraction of bioactive mycoendophyte *N. sphaerica* was spotted on TLC silica gel plates and developed in an optimized solvent system of ethyl acetate/hexane (1:1; *v/v*). TLC sheets were observed under UV light (254 nm) and dried in aseptic conditions. The developed TLC sheets were placed onto sterile Petri plates and overlaid with Mueller-Hinton agar amended with TTC (1 mg/ml) for bacteria and Sabouraud Dextrose agar for fungi previously seeded with 1% standardized (McFarland standard) microbial inoculum and incubated. After incubation, for fungi, the Petri plate was flooded with 10 ml of soft agar (agar 1% *w/v*) amended with MTT (0.05% *w/v)*. Inhibition zones were observed as clear spots, and the active bands and corresponding retention factor (*R*_*f*_) value was measured [19, 23].

#### Antimicrobial activity by disc diffusion method

The disc diffusion method was used to determine the antimicrobial activity of the endophytic fungal extract. Sterile media plates (MHA for bacteria and SDA for fungi) were seeded with predetermined test microbial inocula as described by Rakshith et al. [22]. Sterile discs were impregnated with purified bioactive metabolites from the stock solution (10 mg/ml). The concentration of 30, 60, 150, 300, 450, and 600 μgdisc^-1^ were placed on respective agar media with gentamicin (10 μg/disc; for bacteria) and nystatin (100 U/disc; for fungi) as a positive control, whereas ethyl acetate served as a negative control. Inoculated plates were incubated at 37 ± 2 °C (24 h) for bacteria and 25 °C ± 2 (72 h) fungi. After the incubation, the zone of inhibition was measured around discs in millimeter and results were expressed as mean ± SD.

#### Spectral measurements

##### HPLC profiling

The purity of the bioactive metabolite was analyzed with reference to the chromatogram of the crude ethyl acetate extract of *N. sphaerica* with Shimadzu UFLC – LC – 20 AD series. An amenable 5 μm C18 120 Å, 250 × 4.6 mm LC (A8-ST5C18G120-98) column was used for the detection of metabolites. The injection volume of 20 μL (1 mg/ml) was developed with the mobile phase using methanol with a flow rate of 1 ml/min. The chromatogram was recorded at 260 nm using Lab solutions (Shimadzu corp., Japan) software.

##### LC-MS

The mass of the purified bioactive metabolite was measured using liquid chromatography coupled with Q-TOF in positive electrospray ionization-mass spectrometry (Waters Acquity UPLC Synapt G2 HDMS) with BDS HYPERSIL C18 column (250 × 4.6 mm × 5 μm). The LC separation was recorded using Quattro Premier XE with the Mass Lynx 4.1 software with a gradient solvent system A: 0.1 vol. % formic acid–water and B: 0.1 vol. % formic acid–acetonitrile; 2–98 % B in 50.0 min of run time with a controlled column temperature at 30 °C. The Q-TOF–ESI–MS data were recorded in the range of 200 and 400 nm and positive ESI full MS (60.00–1000.00).

##### NMR

The ^1^H and ^13^C NMR spectra of the purified bioactive metabolite (35 mg/ml) were recorded using deuterated chloroform (d-CDCl_3_) on an Agilent 400-MHz WB (Widerbore) NMR magnet (Santa Clara CA, USA) spectrometer using the VJ3.1 software at 400 MHz and 100 MHz, respectively.

##### FT-IR

Fourier transform infrared spectra of the bioactive purified fraction (1 μLof 1 mg/ml) was recorded using PerkinElmer spectrum TwoTM ATR MIRacle Diamond S2PE (Norwalk, CT, USA) spectrophotometer with the scanning range of 4000–600 cm^-1^.

#### Determination of minimal inhibitory concentration

The MIC was determined by the microdilution method according to Clinical and Laboratory Standards Institute (CLSI) by modifications by [23]. Briefly, the stock solutions of purified phomalactone (10 mg/ml), Gentamicin, and Nystatin (2 mg/ml) were prepared. Two-fold dilutions were carried out in the concentration range of 100–0.19 μg/ml, in 100 μL of sterile Mueller Hinton broth for bacteria and Sabouraud’s dextrose broth for fungi. Twenty microliters of standard suspension of bacterial and fungal inocula were added, except for sterility control well. Gentamicin for bacteria and Nystatin for fungi used as a standard positive control along with growth and sterility controls. Minimal inhibitory concentrations were determined by absorbance at 600 nm after incubation with compounds at 37 ± 2 °C (24 h) for bacteria and 25 °C ± 2 (72 h) fungi [15].

### Statistical analysis

All the experiments were conducted in triplicates and statistical significance such as one-way ANOVA was evaluated using IBM SPSS (Version 25) software.

## Results

### Isolation and identification of endophytic fungi *Nigrospora sphaerica*

**Fig. 1:**
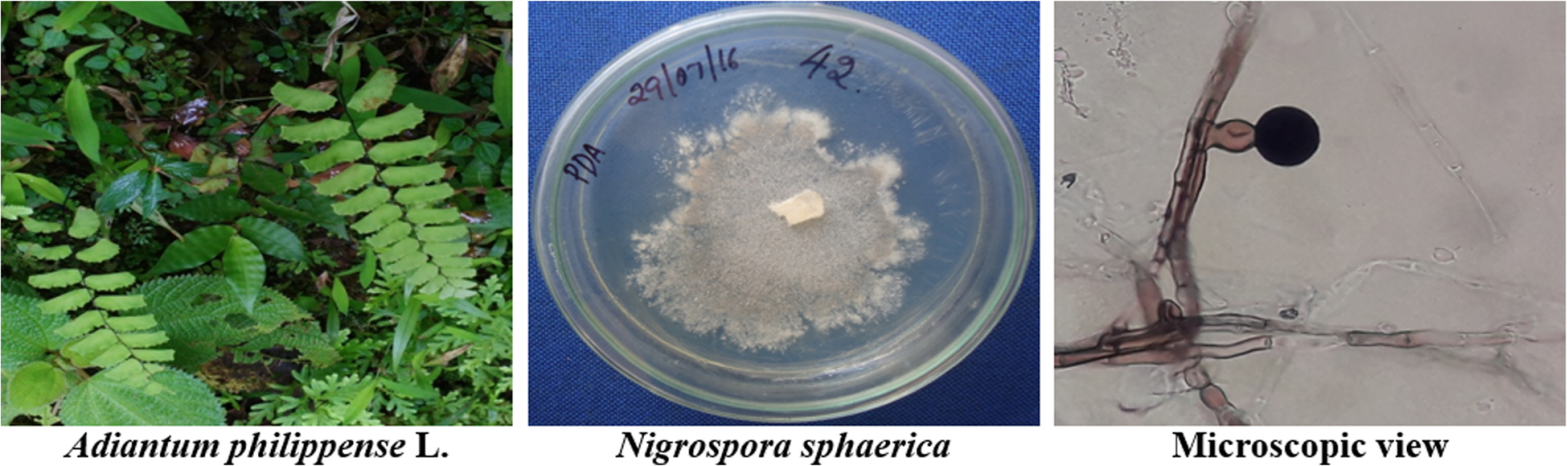
Plant specimen, morphology, and microscopy of *Nigrospora sphaerica*

In the present study, bioactive mycoendophyte isolated from the surface-sterilized leaves of *A. philippense* was subjected to morphological, microscopic (Fig. 1), and molecular identification. Based on ITS rDNA gene sequencing, it was identified as *N. sphaerica* and submitted to GenBank (Accession number: MF400860).

### Phylogenetic affiliation

**Fig. 2:**
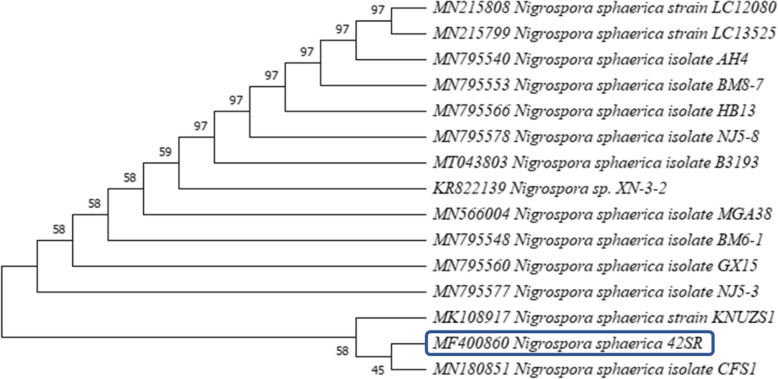
Phylogenetic tree derived from neighbor-joining analysis showing the evolutionary relationship using the MEGA-X software

The amplified sequenced results were processed using Mega X software for assigning putative identity and for assigning OTU’s based similarity. Followed by a neighbor-joining algorithm with a bootstrap value of 1000 for assigning phylogenetic affiliation (Fig. 2).

### Antimicrobial activity of ethyl acetate extract *N. sphaerica*

**Fig. 3:**
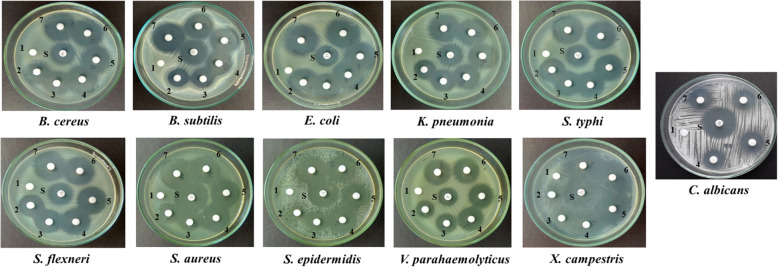
Antimicrobial activity of phomalactone isolated from *N. sphaerica.* NOTE: Standard gentamicin (10 μg disc^-1^; for bacteria) and nystatin (100 U disc^-1^; for fungus) (S), negative control (1), 30 μg disc^-1^ (2), 60 μg disc^-1^ (3), 150 μg disc^-1^ (4), 300 μg disc^-1^ (5), 450 μg disc^-1^ (6), and 600 μg disc^-1^ (7)

The antimicrobial activity of phomalactone from *N. sphaerica* was determined by the disc diffusion assay against a panel of test pathogenic microorganisms. The bioactive compound exhibited an excellent zone of inhibition against all the test microbial pathogens in an increasing order. The antimicrobial activity was found to be highest against *E. coli* with a 24.33 mm diameter followed *S. typhi* (22.67 mm), *B. cereus* (21.67 mm), *S. aureus* (19 mm), *K. pneumonia* (18.67 mm), *B. subtilis* (17.67 mm), *X. campestris* (17.67 mm), *V. parahaemolyticus* (16.67 mm), and* S. epidermidis* (12.67 mm) at 30 μgdisc^-1^ concentration. The metabolite was effective against *Candida albicans* at a higher concentration of 150 μg (Fig. 3, Table 1).

**Table 1:** Antimicrobial activity of phomalactone

Sl. no.	Test organism	Disc diffusion assay							MIC (in μg)	
		Gentamicin (10 μg disc^**-1**^)	30 μg disc^**-1**^	60 μg disc^**-1**^	150 μg disc^**-1**^	300 μg disc^**-1**^	450 μg disc^**-1**^	600 μg disc^**-1**^	Phomalactone	Gentamicin
1.	*Bacillus cereus*	19.33 ± 0.58^f^	21.67 ± 0.58^e^	23.33 ± 0.58^d^	25.33 ± 0.58^c^	26.67 ± 0.58^bc^	28.00 ± 0.00^ab^	29.33 ± 0.58^a^	6.25	0.78
2.	*Bacillus subtilis*	20.33 ± 0.58^de^	17.67 ± 0.58^f^	19.33 ± 0.58^ef^	21.67 ± 0.58^cd^	23.33 ± 0.58^c^	26.33 ± 0.58^b^	31.00 ± 1.00^a^	6.25	0.19
3.	*Escherichia coli*	20.33 ± 0.58^f^	24.33 ± 0.58^e^	25.00 ± 0.00^de^	26.33 ± 0.58^cd^	27.33 ± 0.58^bc^	29.00 ± 1.00^b^	31.67 ± 0.58^a^	3.12	0.39
4.	*Klebsiella pneumonia*	21.33 ± 0.58^d^	18.67 ± 0.58^e^	21.33 ± 0.58^d^	22.67 ± 0.58^d^	25.67 ± 0.58^d^	27.33 ± 0.58^b^	29.33 ± 0.58^a^	6.25	1.56
5.	*Salmonella typhi*	21.00 ± 1.00^f^	22.67 ± 0.58^ef^	23.67 ± 0.58^de^	25.00 ± 0.00^cd^	26.33 ± 0.58^bc^	27.67 ± 0.58^b^	30.00 ± 1.00^a^	6.25	0.39
6.	*Shigella flexneri*	19.67 ± 0.58^f^	20.33 ± 0.58^ef^	22.00 ± 1.00^de^	23.67 ± 0.58^cd^	25.67 ± 0.58^c^	28.00 ± 1.00^b^	31.00 ± 1.00^a^	6.25	1.56
7.	*Staphylococcus aureus*	26.33 ± 0.58^bc^	19.00 ± 1.00^e^	21.67 ± 0.58^d^	24.67 ± 0.58^c^	26.00 ± 0.00^bc^	27.33 ± 0.58^b^	31.00 ± 1.00^a^	12.5	0.19
8.	*Staphylococcus epidermidis*	27.67 ± 0.58^a^	12.67 ± 0.58^d^	17.67 ± 0.58^c^	22.67 ± 0.58^b^	24.67 ± 0.58^b^	27.00 ± 1.00^a^	29.00 ± 1.00^a^	12.5	0.19
9.	*Vibrio parahaemolyticus*	17.33 ± 0.58^de^	16.67 ± 0.58^e^	18.67 ± 0.58^d^	22.33 ± 0.58^c^	24.33 ± 0.58^b^	25.67 ± 0.58^ab^	26.67 ± 0.58^a^	6.25	0.78
10.	*Xanthomonas campestris*	30.00 ± 1.00^b^	17.67 ± 0.58^e^	19.33 ± 0.58^e^	22.00 ± 1.00^d^	24.67 ± 0.58^c^	31.00 ± 1.00^b^	33.67 ± 0.58^a^	3.12	0.39
11	*Candida albicans*	25.67 ± 0.58^a^	NA	NA	16.33 ± 0.58^d^	17.33 ± 1.15^cd^	18.67 ± 0.58^bc^	20.33 ± 0.58^b^	12.5	0.19

Values followed by the same superscript letter(s) are significantly different at (*p <* 0.05) by Tukey’s post hoc test

*NA* no activity

### TLC-bioautography assay

**Fig. 4:**
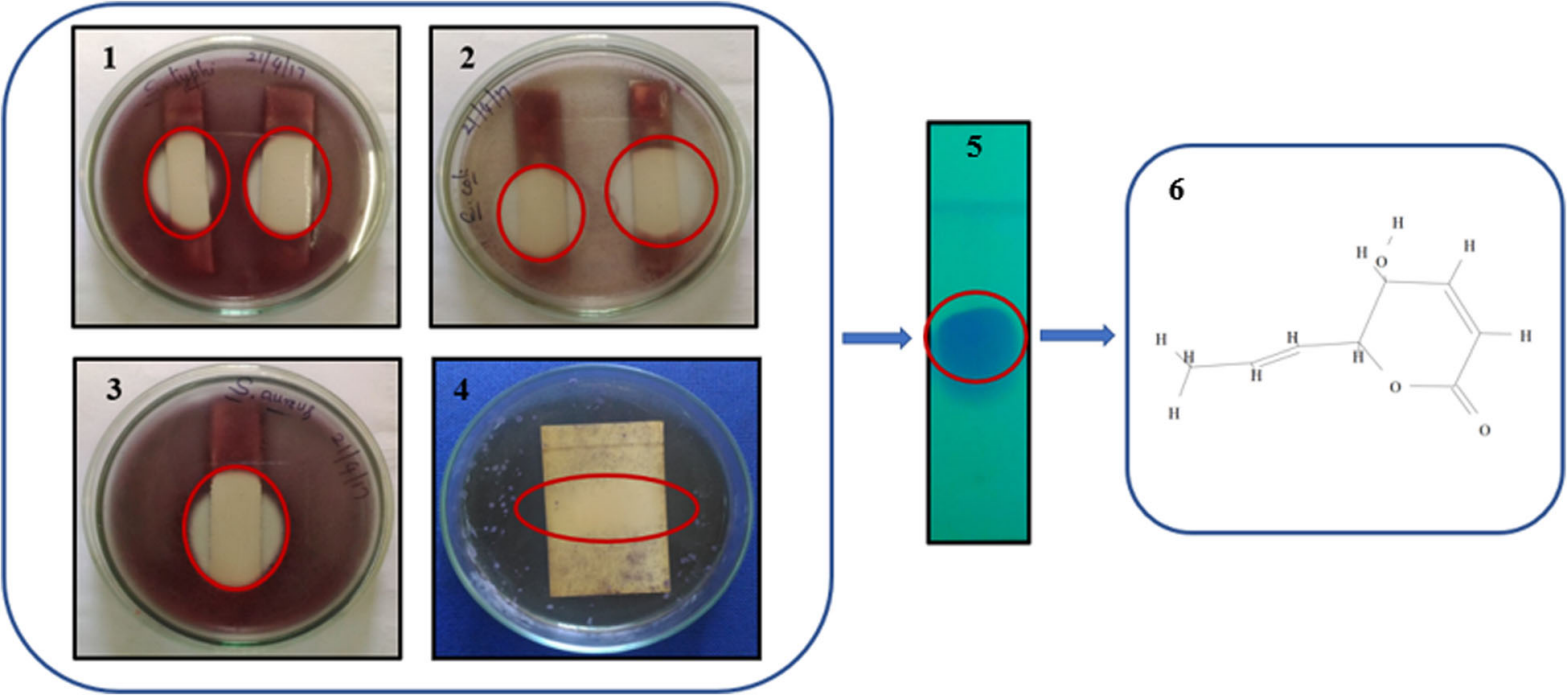
TLC bioautography assay of purified phomalactone against selected microbial pathogens. (1) *S. typhi*, (2) *E. coli*, (3) *S. aureus*, (4) *C. albicans*, (5) TLC chromatogram (under UV light), and (6) structure of phomalactone

Metabolite with antimicrobial activity was identified as a clear zone against a red background on Mueller-Hinton agar plates and a dark blue background on the Sabouraud Dextrose agar plate. The zone of inhibition was observed at a *R*_*f*_ = 4.5 indicating the purified bioactive metabolite from the ethyl acetate extract of *N. sphaerica* (Fig. 4).

### Spectral data

**Color**: Yellow

**Physical state:** Amorphous Semi-Solid

**Yield:** 0.45 g/L

**Name:** Phomalactone [(5R, 6R)-5 hydroxy-6-[(E)-prop-1-en-1-yl]-5, 6-dihydroxy-2 h-pyran-2-one].

**HPLC:** The purified compound eluted at RT-4.89 with 86% purity (Supplementary Figure 1).

**LCMS:** 155.126 (positive mode) (Supplementary Figure 2)

**NMR:**

^**1**^**H NMR:** (400 MHz, CDCl_3_-d6, ℧, ppm): 2.05 (s, 3H), 2.80 (s, 1H, –OH), 4.53 (m, 1H), 4.76 (m, 1H) 5.67–5.69 (dd, or q, 2H), 6.03–6.04 (m, 2H) (Supplementary Figure 3).

^**13**^**C NMR:** (100 MHz, CDCl_3_-d6, ℧, ppm): 17.6 (C-9), 63.8 (C-5), 122.3 (C-3), 132.7 (C-8), 144.8 (C-4), 162.1 (C-2, C-0), 83.3 (C-6), 123.5 (C-7). MS (ESI, m/z): 154.06, (100%), 155.07 (M + 1) (Supplementary Figure 4).

**FT-IR:** 3600-3200 (S, –OH stretch), 1750–1720 (ether), 1680–1600 (alkene), 1448 (CH_3_-bond), 1050–1150 (> C=O (1160) stretch), 1380 (–CH-bond) (Supplementary Figure 5).

The compound obtained was in the form of a yellow amorphous semi-solid with a molecular mass of 155.07 in ESI-positive mode. The ^1^H NMR spectrum revealed the presence of a secondary methyl group attached to carbon 5 in the compound. The ^13^C NMR revealed 8 carbon signals: signals for one quaternary carbon atoms, 6 methane groups, and one methyl group representing dihydropyranon chemical skeleton. The presence of the hydroxyl group at 3600–3200 cm^-1^ and carbonyl at 1160 cm^-1^ confirmed the bioactive to be phomalactone.

### Minimal inhibitory concentration

The MIC was found to be lowest against *E. coli* and *X. campestris* at 3.12 μg concentration followed by *S. typhi*, *B. subtilis*, *B. cereus*, and *K. pneumonia* at 6.25 μg. The inhibition was observed at 12.5 μg for *S. aureus*, *S. epidermidis*, and *C. albicans* (Table 1**)**. The results obtained indicated that Gram-negative bacteria were found to be more susceptible when compared to Gram-positive.

## Discussions

*Nigrospora sphaerica* has also been reported from a vast range of host viz., *Ginkgo biloba* [20], *Moringa oleifera* [38], *Phoenix dactylifera* [1] and *Saccharum arundinaceum* [36]. In this study, the secondary metabolites of *N. sphaerica*, extracted with ethyl acetate showed a significant antimicrobial activity against bacteria and fungus viz., *S. aureus*, *B. subtilis*, *S. epidermidis*, *E. coli*, *K. pneumonia*, *P. aeruginosa*, *S. typhi*, *S. flexneri*, *X. campestris*, *V. parahaemolyticus*, and *C. albicans*, respectively. Several bioactive compounds have been reported from *N. sphaerica* which suggests the potential for discovery of pharmaceutically relevant substances. Previously, it was reported *N. sphaerica* to produce new antimicrobial bioactive secondary metabolites, such as nigrosporolides [5], nigrosporins [28], lactones [4], epoxydons and pyrones, diterpenes, diketopiperazines [3], and nigrosporolides [37].

Phomalactone is quite insecticidal [14] and has been isolated previously from several species of the fungal genera *Nigrospora* [30, 33], *Xylaria* [6], *Aspergillus* [11], *Verticillium* [7], *Drechslera* [13], *Hirsutella* (entomopathogenic) [12], *Paecilomyces* (entomopathogenic) [32], and *Phoma* [34]. Phomalactone has antifungal activity against *Aspergillus fumigatus* [11], nematocidal activity [7], immunomodulating activity [13], insecticide activity [12], and phytotoxic activity [4].

Phomalactone from endophyte *Xylaria* sp. exhibited to possess weak activity at 13 μg/ml against protozoan *Plasmodium falciparum* was reported by Carlos et al. 2008. A study conducted by Kumudini et al. 2015 reported phomalactone from *N. sphaerica* had larvicidal, adulticide activity against *Aedes aegypti* and *Anopheles quadrimaculatus* mosquitoes with LD_50_ value 0.64 μg/org and 0.20 μg/org, respectively. The investigation of biopotential endophytes inhabiting *Zoysia japonica* Steud by Kim et al. 2001 revealed phomalactone isolated from *N. sphaerica* exhibited potent fungitoxic activity against *Phytophthora infestans* and *Phytophthora capsici*. Its ecological role(s) in each of these species has not been rigorously studied, but its phytotoxic, fungitoxic, and insecticidal activities may be important to the various fungi that produce phomalactone.

## Conclusion

In conclusion, this is the first report on the isolation of *N. sphaerica* from medicinal plant *A. philippense*. Chemical investigation through bioautography *N. sphaerica* displayed a broad spectrum of antimicrobial activity, which indicated that endophytic *N. sphaerica* as a potent producer of bioactive phomalactone derivative with great potential as a natural product drug molecule.

## Supplementary information

**Additional file 1: Supplementary Figure 1.** HPLC analysis of purified bioactive compound phomalactone.

**Additional file 2: Supplementary Figure 2.** LCMS spectra of purified bioactive compound phomalactone.

**Additional file 3: Supplementary Figure 3.** Proton (^1^H) NMR Spectra of purified bioactive compound phomalactone.

**Additional file 4: Supplementary Figure 4.** ^13^C NMR Spectra of purified bioactive compound phomalactone.

**Additional file 5: Supplementary Figure 5.** FT-IR Spectra of purified bioactive compound phomalactone.

## Data Availability

The data from the study is publicly available.

## Abbreviations

Dw: Distilled water
PDA: Potato dextrose agar
PDB: Potato dextrose broth
MHA: Mueller-Hinton Agar
TLC: Thin layer chromatography
TTC: 2,3,5-Triphenyltetrazolium chloride
MTT: 1-(4,5-Dimethylthiazol-2-yl)-3,5-diphenylformazan, Thiazolyl blue formazan
SDA: Sabouraud dextrose agar
MIC: Minimal inhibitory concentration
