# Supplementary figures and images for: Antimicrobial metabolite profiling of Nigrospora sphaerica from Adiantum philippense L
Source: J Genet Eng Biotechnol. 2020 Oct 22;18:66. doi: 10.1186/s43141-020-00080-4 (PMC7581665; doi:10.1186/s43141-020-00080-4)

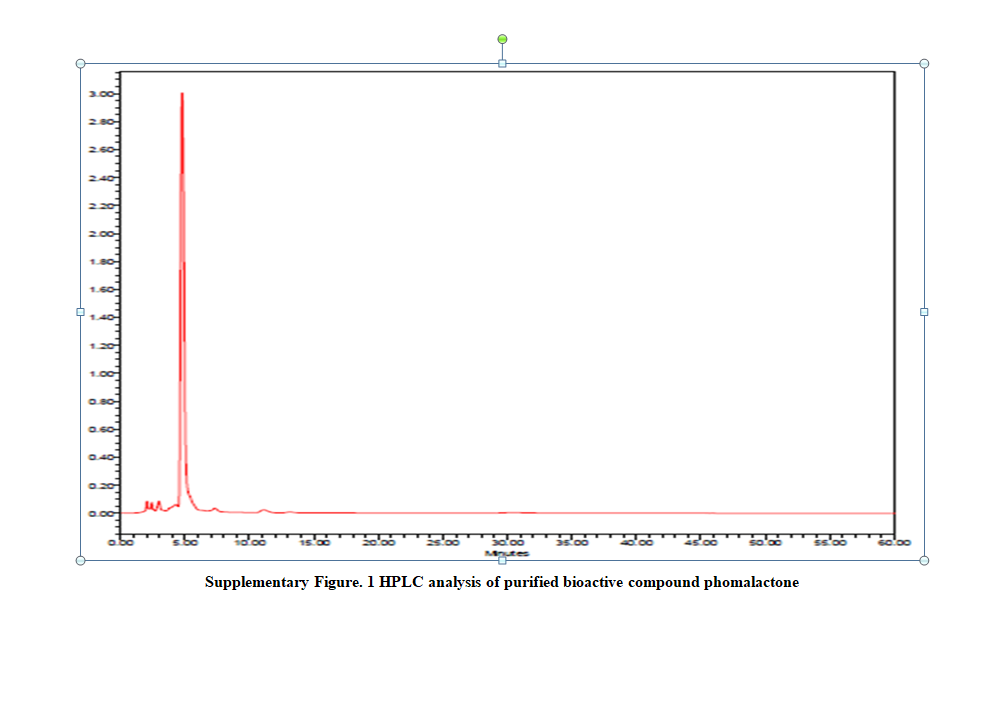

Supplement: Supplementary file 1 — Additional file 1: Supplementary Figure 1. HPLC analysis of purified bioactive compound phomalactone. [file 43141_2020_80_MOESM1_ESM.png]

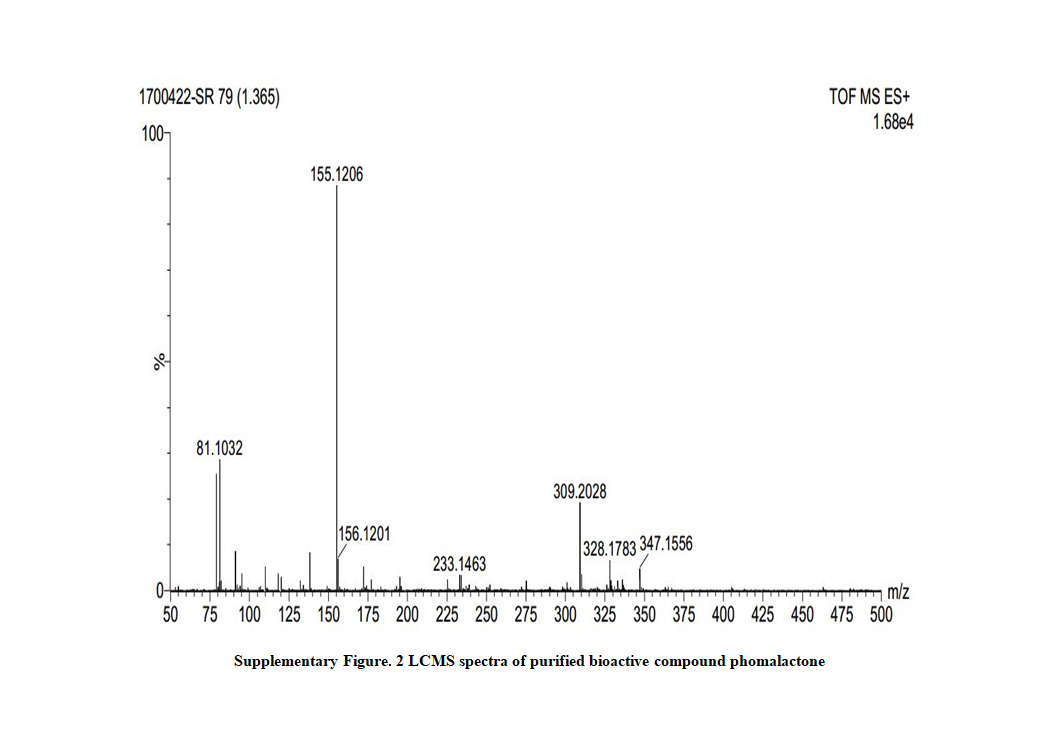

Supplement: Supplementary file 2 — Additional file 2: Supplementary Figure 2. LCMS spectra of purified bioactive compound phomalactone. [file 43141_2020_80_MOESM2_ESM.png]

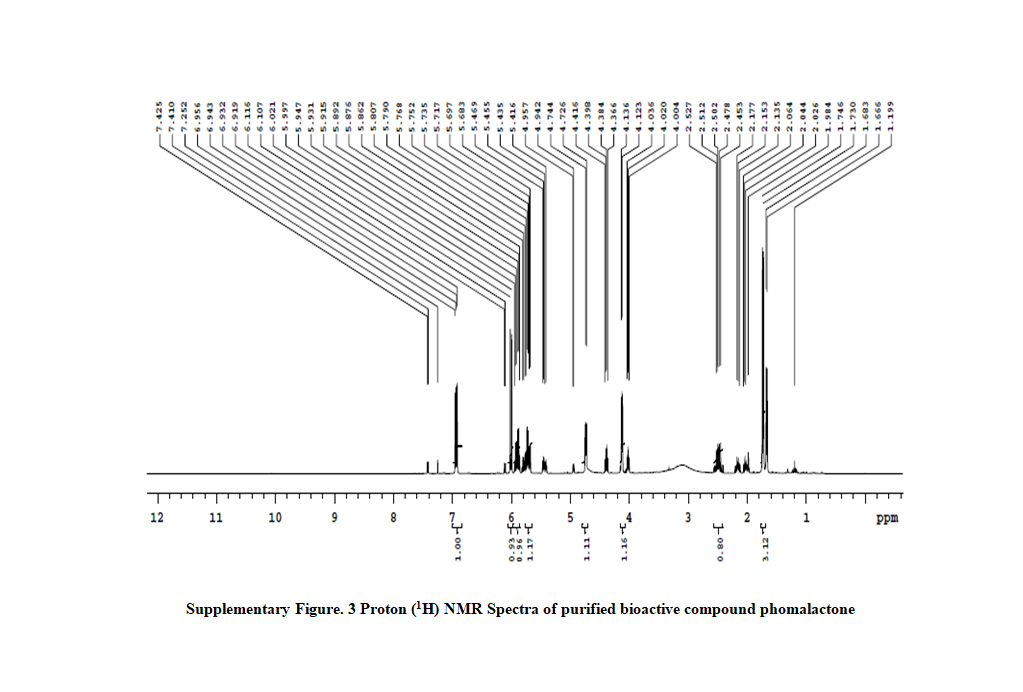

Supplement: Supplementary file 3 — Additional file 3: Supplementary Figure 3. Proton (1H) NMR Spectra of purified bioactive compound phomalactone. [file 43141_2020_80_MOESM3_ESM.png]

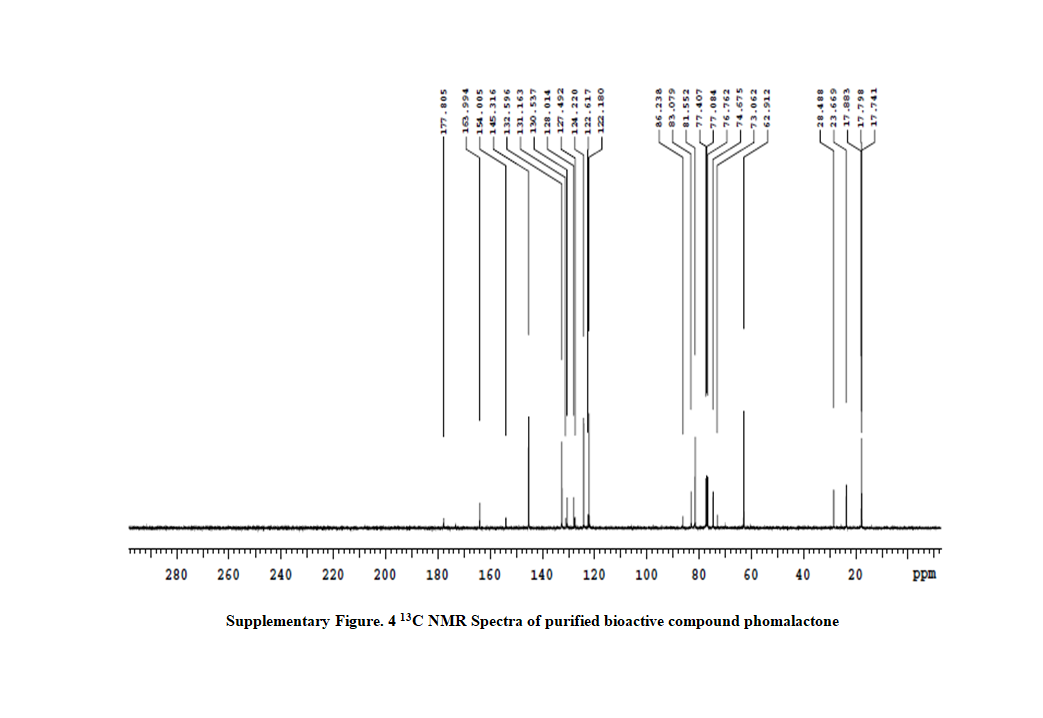

Supplement: Supplementary file 4 — Additional file 4: Supplementary Figure 4. 13C NMR Spectra of purified bioactive compound phomalactone. [file 43141_2020_80_MOESM4_ESM.png]

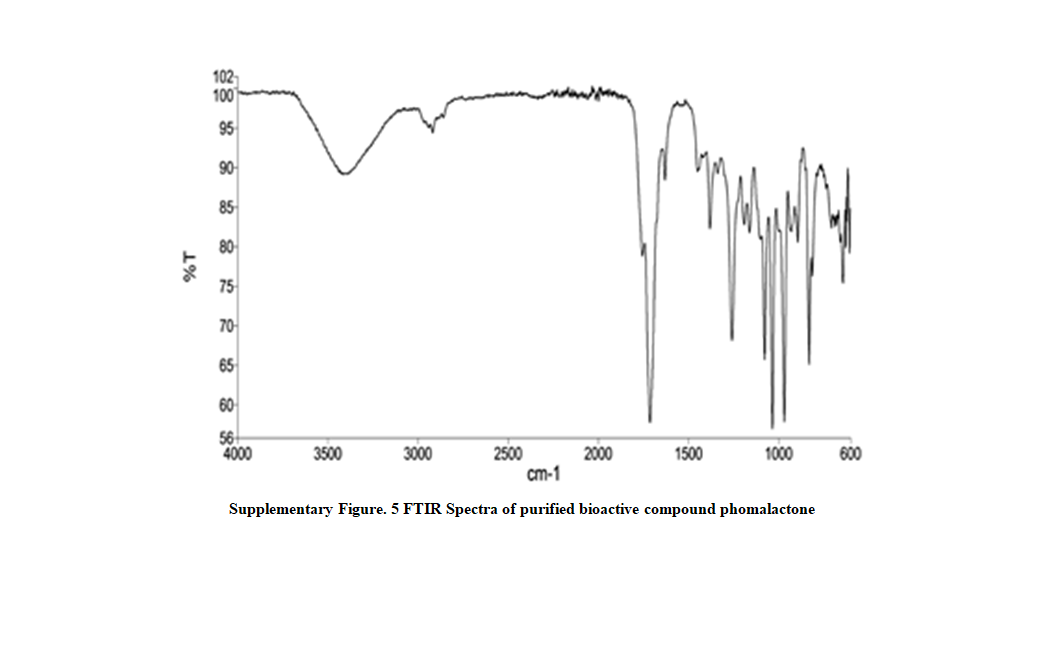

Supplement: Supplementary file 5 — Additional file 5: Supplementary Figure 5. FT-IR Spectra of purified bioactive compound phomalactone. [file 43141_2020_80_MOESM5_ESM.png]
